# *Tropheryma whipplei* Genotypes 1 and 3, Central Europe

**DOI:** 10.3201/eid1902.120709

**Published:** 2013-02

**Authors:** Nils Wetzstein, Florence Fenollar, Sylvain Buffet, Verena Moos, Thomas Schneider, Didier Raoult

**Affiliations:** Author affiliations: Université Aix-MarseilleMarseilles, France (N. Wetzstein, F. Fenollar, S. Buffet, D. Raoult);; Charité Universitätsmedizin Berlin, Berlin, Germany (N. Wetzstein, V. Moos, T. Schneider)

**Keywords:** Whipple disease, Tropheryma whipplei, bacteria, bacterial genotyping, central Europe, Marseilles, France

**To the Editor:** *Tropheryma whipplei* causes Whipple disease, a rare multisystemic disorder that affects mainly middle-aged white men and is most widely distributedin Europe and North America (*1*). In the general population of France, *T. whipplei* DNA was found in 2%–4% of stool samples and *T. whipplei*–specific antibodies were found in 51% of serum samples (*2*). Still, the prevalence of classic Whipple disease, which causes arthralgia, diarrhea, and weight loss, remains extremely low (*1*). Whipple disease has 4 known manifestations: 1) classic Whipple disease; 2) focused chronic infections, mainly endocarditis; 3) acute infections, such as gastroenteritis, bacteremia, and pneumonia; and 4) asymptomatic *T. whipplei* carriage in healthy persons (*1**–**5*). *T. whipplei* is thought to be transmitted through oral and oro–fecal routes by human-to-human contact (*2**,**6*).

The pathogen was cultivated in 2000, and 2 genomes were sequenced (reference strains Twist and TW08/27) (*7**,**8*). These events made possible a genotyping system based on 4 highly variable genetic sequences found by genome comparison (TW133, ProS, SecA, Pro184) (*9*). Since 2007, we have applied this system to patient samples positive for *T. whipplei* from central Europe and sub-Saharan Africa (*2**,**3**,**9**,**10*). The system showed a higher discriminatory power than previous typing methods and improved the genotyping resolution of *T. whipplei*, promoting better understanding of its epidemiology on the molecular level (*9*).

Since 2003, we have subcultured strain Twist every 3 weeks. In 2007 and 2012, we compared sequences for the subcultured strains with that for the 2003 strain. We found that the spacer sequence remained stable over the ≈10-year period. This finding suggests a high intrastrain genetic stability and highlights the value of the typing system, which is stable. Thus, a change in genotype in a patient with Whipple disease must be interpreted as an infection with a different strain and cannot be attributed to mutation of the original strain.

To date, 191 samples positive for *T. whipplei* collected from patients from central Europe (France, Germany, Switzerland, Austria, and Italy) have been typed, revealing a genetic diversity by identifying 72 different *T. whipplei* genotypes: 1–23, 25–45, 58–60, 76–77, 82–102, and 111–116. The discriminatory power was high (Hunter-Gaston discriminatory index 0.9298) for all samples from Europe. No connection between clinical manifestations and *T. whipplei* genotypes has been described. Reasons might be found either in an unknown link between genomics in *T. whipplei* and clinical outcome or might be because the typing system cannot identify possible virulence factors. Genotypes 1 and 3 are predominant (*1*,*3*), accounting for 35.1% of all tested *T. whipplei* samples from Europe.

Genotype 3 is the most common *T. whipplei* genotype in Europe (19.9% of all samples) and could be considered epidemic in and specific to France, Switzerland, and Italy. This genotype was proposed to be responsible for small outbreaks caused by clonal strains, such as gastroenteritis among young children or the strain carried by homeless persons in Marseille, France (*3**,**6*), but it has not been described in Germany or Austria (Figure). 

**Figure:**
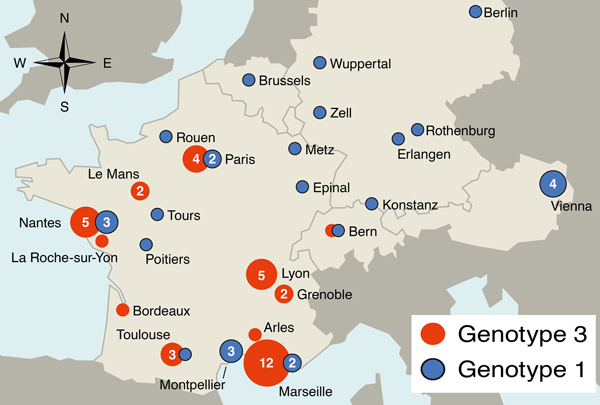
Geographic distribution of the 2 most common genotypes of *Tropheryma whipplei* in central Europe. Numbers in circles indicate number of cases with corresponding genotype; circles without a number indicate single cases. Cities are either the residence of patients or of their physicians; the capital of the country of residence is shown for persons whose city of residence was unknown. This map was made by using Epi Info 7 (wwwn.cdc.gov/epiinfo/).

Genotype 1 is found throughout central Europe and is the second most common genotype (15.2% of all samples) (Figure). Predominance of this genotype in Germany is high (46.2%, n = 13) and Austria (80%, n = 5). Infection with this genotype seems to be endemic in the population of the area, although no outbreaks have been reported. 

Of 191samples, a total of 55 (28.8%) showed a unique genotype consistent with the high genetic variability in *T. whipplei* specimens. Phylogenetic analysis and clustering of these singletons showed no correlation between clusters and geographic origin of samples.

Of the 191 samples, a total of 66 (34.6%) were from Marseille and represented 40 different genotypes. This finding underscores the broad heterogeneity in *T. whipplei*. Twelve (18.5%) of the 66 tested samples were genotype 3, which might be linked to the local outbreak among homeless persons mentioned above. Genotype 1, which is endemic to France, was found in only 2 (3.1%) persons in Marseille. The fact that Marseille is a metropolitan area with a high migration rate could play a role in the vast diversity of *T. whipplei* genotypes found there

Questions regarding the epidemiologic character of Whipple disease remain unanswered, such as why the bacterium is highly prevalent but the disease is not. Persons with the putative immunological defect probably responsible for classic Whipple disease (*1*) have the highest bacterial load in their stools. But these persons are unlikely to come into direct contact with one another. Thus, propagation of this bacterium on a large scale might be relatively limited, which could explain the high genetic diversity in the bacterial specimens assessed so far.

Two predominant genotypes seem to break out of this pattern: genotypes 1 and 3. Genotype 3 could be considered a genotype that causes small epidemics, whereas genotype 1 could be considered a genotype endemic to central Europe. Reasons for the success of these 2 genotypes remain unknown, but improvement of genotyping methods could provide the answers.
